# Extended genotype–phenotype spectrum of 17α-hydroxylase/17,20-lyase deficiency: a nine-case series featuring a novel mutation, suspected TART-like lesions, and multisystem involvement

**DOI:** 10.3389/fendo.2026.1824733

**Published:** 2026-09-17

**Authors:** Hemeng Chong, Yutong Fu, Xuan Zhang, Yanan Zhang, Xue Yan, Yalei Pi

**Affiliations:** 1 Department of Pediatrics, the Second Hospital of Hebei Medical University, Shijiazhuang, China; 2 Department of Pathology, the Second Hospital of Hebei Medical University, Shijiazhuang, China

**Keywords:** 17α-hydroxylase/17,20-lyase deficiency, congenital adrenal hyperplasia, CYP17A1 gene, novel mutation, testicular adrenal rest tumor

## Abstract

**Context:**

17α-hydroxylase/17,20-lyase deficiency (17-OHD), a rare congenital adrenal hyperplasia driven by biallelic CYP17A1 variants, shows extensive clinical and molecular heterogeneity; data on rare phenotypes and genotype–phenotype patterns are scarce.

**Objective:**

To characterize clinical, hormonal, gonadal pathological, and genetic features of nine Chinese patients with 17-OHD from a single center, expand the population-specific CYP17A1 variant spectrum, explore genotype–subtype trends, and document rare incidental manifestations to inform clinical care and genetic counseling.

**Methods:**

A retrospective case series included nine genetically verified patients (seven 46,XY, two 46,XX). SWISS-MODEL homology modeling and American College of Medical Genetics and Genomics (ACMG) criteria were applied to assess the novel variant’s pathogenicity.

**Results:**

The homozygous c.985_987delTACinsAA (p.Y329Kfs^*^90) variant predominated in complete-type patients. A novel nonsense variant c.1218G>A, p.W406X, predicted to truncate 103 C-terminal amino acids, was tentatively classified as pathogenic based on PVS1/PM2/PM3/PM4 evidence in one partial-type case. We observed a descriptive trend: biallelic truncating loss-of-function variants correlated with complete 17-OHD, while partial forms carried missense variants with residual enzyme activity. The two 46,XX complete-type patients had bilateral ovarian atrophy and low anti‑Müllerian hormone (AMH); two 46,XY patients presented with incidental unilateral sensorineural hearing loss. One 46,XY patient harbored gonadal steroidogenic cell nests resembling testicular adrenal rest tumors (TART), without adrenal-specific immunohistochemistry (IHC) to confirm cellular origin. One proband had two independent monogenic diseases: 17-OHD and left ventricular non-compaction cardiomyopathy, carrying concurrent CYP17A1 and sarcomeric gene (MYBPC3/ACTN2/TTN) variants. All patients had elevated adrenocorticotropic hormone (ACTH), most had low cortisol, and 11-deoxycorticosterone (11-DOC) and corticosterone levels were variably elevated.

**Conclusions:**

This cohort extends the Chinese pathogenic CYP17A1 variant spectrum and reveals tentative genotype–subtype associations plus rare co-occurring phenotypes. Larger multicenter cohorts and functional experiments are needed to validate causal links between atypical manifestations and 17-OHD. Early adrenal steroid profiling and CYP17A1 sequencing are recommended for patients with hypertension, hypokalemia, and disorders of sex development. Serial ovarian reserve testing is required for 46,XX patients, and long-term gonadal imaging follow-up for 46,XY patients with gonadal dysgenesis.

## Introduction

17-OHD is a rare form of congenital adrenal hyperplasia (CAH) caused by mutations in the *CYP17A1* gene, with an estimated prevalence of 1–9 per 1,000,000. “Founder effects” have been described in China and Brazil (1, 2). The enzyme 17α-hydroxylase/17,20-lyase, encoded by *CYP17A1* and expressed in both gonads and adrenal glands, is a key bifunctional enzyme in steroidogenesis: it catalyzes the 17α‑hydroxylation of pregnenolone and progesterone to yield 17α‑hydroxylated products, followed by 17,20‑bond cleavage to generate C19 androgen precursors (3). Consequently, deficiency of this enzyme impairs the synthesis of both cortisol and sex steroids.

17-OHD biochemically features impaired cortisol production, elevated adrenocorticotropic hormone (ACTH), and buildup of the mineralocorticoid precursor 11-deoxycorticosterone (11-DOC). Its phenotypes vary widely: 46,XY patients show genital undervirilization, while 46,XX patients suffer primary amenorrhea and failed sexual maturation (4–6). Excess 11-DOC causes hyporeninemic hypertension and hypokalemia. Patients with all typical symptoms are defined as having complete 17-OHD; partial 17-OHD refers to 46,XY individuals with ambiguous genitalia and 46,XX patients with incomplete puberty, who may also develop hypertension and hypokalemia (7, 8).

This retrospective analysis of nine 17-OHD patients systematically characterizes clinical, hormonal, and *CYP17A1* variant profiles to propose tentative genotype–phenotype trends. We describe incidental suspected testicular adrenal rest tumors (TART)-like steroidogenic nests and unilateral hearing loss, alongside a novel pathogenic variant, providing novel insights for clinical practice, genetic counseling, and mechanistic research.

## Materials and methods

### Study subjects

All patients were initially suspected to have 17-OHD based on pathognomonic clinical phenotypes (hypertension, hypokalemia, disorders of sex development, delayed puberty). Six patients completed full steroid profiling including measurements of 11-DOC, corticosterone, renin, and aldosterone with consistent biochemical evidence for 17-OHD. The other three received only basic cortisol, ACTH, and sex hormone tests without complete steroid panels; their diagnoses were ultimately confirmed by CYP17A1 sequencing.

### Clinical and biochemical data collection

We collected and analyzed the following data for all patients: chief complaint, age at diagnosis, external genitalia phenotype, blood pressure, serum electrolytes, ACTH, cortisol, follicle‑stimulating hormone (FSH), luteinizing hormone (LH), progesterone (P), 17‑hydroxyprogesterone (17OHP), testosterone (T), 11‑DOC, and corticosterone levels. anti‑Müllerian hormone (AMH) and inhibin B levels (if available), bone age (BA), pelvic ultrasound, and gonadal histopathology findings were also recorded.

### Genetic testing

Peripheral blood DNA from all probands and available parents was extracted for familial co-segregation analysis. Eight patients underwent targeted sequencing covering 276 CAH-related genes on the Illumina NovaSeq 6000 platform, while the P6 pedigree received whole-exome sequencing on the MGI DNBSEQ-T7 platform. Libraries were constructed and enriched with the Agilent SureSelect kit. Raw reads underwent quality control, alignment, variant calling, and ANNOVAR annotation; ExomeDepth was used for copy-number variant detection. Mean target depth reached ≥200×, with ≥100× coverage across 98.7% of core exons.

All variants were annotated according to Human Genome Variation Society (HGVS) standards using the following reference transcripts: CYP17A1 NM_000102.4, MYBPC3 NM_000256.3, ACTN2 NM_001103.3, and TTN NM_133379.5. Pathogenicity was assessed via the American College of Medical Genetics and Genomics (ACMG) criteria (9, 10), public databases, and InterVar software. All biallelic CYP17A1 variants were validated using ABI 3730 Sanger sequencing to confirm autosomal recessive segregation.

### *CYP17A1* homology modeling

To assess the structural impact of newly identified mutations, homology modeling was performed using the SWISS‑MODEL server (https://swissmodel.expasy.org/) with the wild‑type *CYP17A1* crystal structure (template: 6chi.3.A) as a reference. The resulting three‑dimensional model was visualized and analyzed using PyMOL (https://pymol.org/2/) to evaluate potential structural alterations and predict structural consequences of novel variants.

## Results

### Basic clinical characteristics

Nine patients were enrolled, with clinical data listed in **Table 1**. All exhibited cutaneous (and genital) hyperpigmentation; seven had complete 17-OHD. Four 46,XY patients (P1, P3, P4, P8) developed hypertension and had full female genitalia in childhood or adolescence. Three of them received laparoscopic gonadectomy at 9.7–18.1 years, and histology confirmed testicular dysgenesis. Two 46,XX patients (P2, P9) presented with hypertension or absent puberty; ultrasound showed a detectable uterus and undetectable ovaries, and AMH levels were reduced. P3 and P4 additionally had left-sided sensorineural hearing loss.

**Table 1:** Clinical characteristics of nine patients with 17-OHD.

Variables	P1	P2	P3	P4	P5	P6	P7	P8	P9
Chief Complaint	No secondary sexual development	Hypertension	Hypertension	No secondary sexual development	Ambiguous genitalia.	Hypertension	Ambiguous genitalia.	Hypertension	No secondary sexual development
Diagnosis Age (yr)	14.6	13.2	9.0	17.8	0.2	10.2	17.6	15.6	15.7
Chromosome karyotype	46,XY	46,XX	46,XY	46,XY	46,XY	46,XY	45,XY,der(13;14)(q10;q10)	46,XY	46,XX
phenotype	female	female	female	female	undervirilized	female	undervirilized	female	female
BP (mm Hg)	148/89	141/96	140/90	156/122	NA	161/114	102/74	174/95	115/65
CA-BA (y)	3.4	4.4	1.5	6.3	NA	3.1	NA	5.3	6.2
K (mmol/L)	2.83	3.30	4.22	2.62	3.12	3.43	3.34	3.66	3.88
ACTH (pg/mL)	>1250.0	78.4	354.0	118.3	114.8	>1250.0	215.3	>1250.0	154.7
Cortisol (ng/mL)	38.7	14.6	5.6	15.9	24.1	9.6	55.4	5.2	7.1
FSH (mIU/mL)	90.9	56.3	20.8	108	NA	24.0	30.5	117	71.7
LH (mIU/mL)	31.9	18.0	9.6	36.2	NA	5.4	29.2	55.0	35.6
P (ng/mL)	10.7	13.6	6.8	12.3	0.6	1.4	2.7	14.8	7.44
17OHP (ng/mL)	ND	ND	0.1	0.3	0.1	ND	1.9	ND	ND
T (pg/mL)	10.6	ND	ND	20	14.6	0.7	529.7	ND	2.1
11-DOC (pg/mL)	2588.8	NA	NA	NA	368.0	1439.0	585.2	722.3	551.6
Corticosterone (ng/mL)	191.9	NA	NA	NA	102.4	110.7	91.1	116.4	43.9
AMH (ng/mL)	NA	2.9	NA	NA	NA	NA	NA	NA	2.0
Genotype	p.I259Hfs^*^16/p.R96W	p.Y329Kfs^*^90/p.Y329Kfs^*^90	p.Y329Kfs^*^90/p.Y329Kfs^*^90	p.Y329Kfs^*^90/p.Y329Kfs^*^90	p.L433S/ p.168_169delSFinsF	p.Y329Kfs^*^90/ p.Y329Kfs^*^90	p.W406X/ p.I332T	p.R96Q/ p.Y329Kfs^*^90	p.Y329Kfs^*^90/ p.Y329Kfs^*^90
Gonadal Ultrasound	No uterus or ovaries detected	Primordial uterus, bilateral ovaries not detected	No uterus or ovaries detected; bilateral hypoechoic areas around iliac vessels. Bilateral inguinal hernias	No uterus, ovaries, or vagina detected	NA	No uterus or ovaries detected; bilateral heterogeneous echoes in inguinal regions	No uterus or ovaries detected; bilateral hypoechoic nodules adjacent to pubic symphysis	No uterus or ovaries detected; hypoechoic nodules in inguinal region	Small uterus, bilateral ovaries unclear
Adrenal volume	Bilateral hyperplasia	Mild thickening of left adrenal body	Left adrenal thickening	NA	NA	NA	Normal morphology	Bilateral hyperplasia	NA
Gonadectomy Age (y)	15.2	--	9.7	18.1	4.0	10.4	24.7	Not done	--
Histopathology^a^	dysplastic testicular tissue	--	dysplastic testicular tissue	dysplastic testicular tissue with multifocal calcification	Undeveloped testicular tissue	Bilateral testicular tissue with multifocal calcification; left side shows a small amount of adrenal tissue	Testicular seminiferous tubule dysgenesis, Sertoli cells visible, tubule atrophy, and sclerosis	NA	--
Other System Anomalies	None	None	Left-sided sensorineural hearing loss	Left-sided sensorineural hearing loss	None	Left ventricular non-compaction	None	None	None

BP, blood pressure. CA-BA, the difference between chronological age and bone age; yr, year; y, year. NA, not available. ND, Not Detected (below limit of quantification). a: Archived pathological slides without standardized detailed microscopic records due to data limitations.

Two patients (P5, P7) presented with the partial form, both with an XY karyotype and undervirilized external genitalia. P5 was identified in infancy due to clitoromegaly and labial abnormalities with a palpable mass, leading to removal of undeveloped testicular tissue at 4 years. P7 presented in adolescence with absent breast development and menarche; examination revealed clitoromegaly and labial fusion. Cytogenetic analysis showed a 45,XY,der (13, 14)(q10;q10) karyotype, indicative of a Robertsonian translocation. The patient underwent bilateral cryptorchidectomy and vulvoplasty at 24.7 years.

Patient P6 had a distinct clinical course. Initially evaluated in childhood for non-compaction cardiomyopathy, the patient was diagnosed with hypertension at 10 years of age, which subsequently led to the definitive diagnosis of 17-OHD. The patient exhibited a female phenotype. Ultrasonography detected neither uterine nor ovarian tissues. Chromosomal analysis confirmed a 46,XY karyotype. Histopathology of the excised gonads showed bilateral testicular dysgenesis with multifocal calcification. A small focus of adrenal tissue within the left gonad was consistent with a suspected TART-like lesion (**Figure 1**). Family history revealed that the patient’s mother had echocardiographic evidence of non‑compaction cardiomyopathy but remained asymptomatic, while the father was healthy.

**Figure 1:**
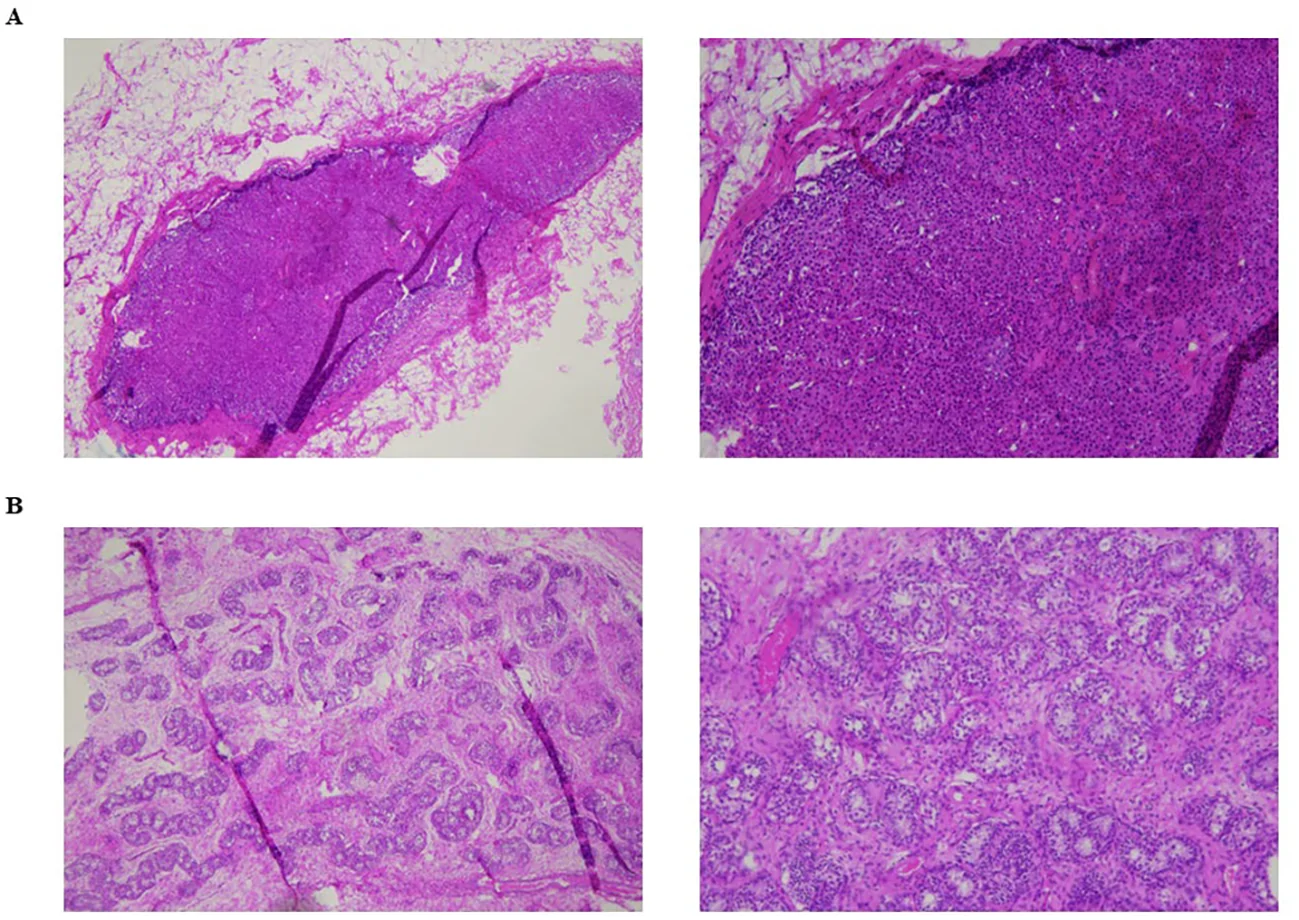
Hematoxylin and eosin (H&E)-stained gonadal histopathology of patient P6. **(A)** Suspected TART-like foci in dysplastic left testis (25× and 40× magnification). Without adrenal-specific immunohistochemistry (CYP11B2, calretinin), these lesions cannot be distinguished from gonadotropin-triggered Leydig cell hyperplasia and are thus only suspected TART-like lesions. **(B)** Dysplastic testicular tissue with multifocal calcification (25× and 40× magnification).

### Hormonal and imaging analysis

All patients had elevated ACTH (9/9), with cortisol levels below the normal range in eight cases. Most showed raised FSH, LH, and progesterone alongside reduced 17OHP. 11-DOC and corticosterone were significantly elevated (6/6). Adrenal volume was enlarged (4/5) or normal (1/5). BA was delayed to varying degrees in all seven patients assessed.

### Genetic characteristics

Among the seven complete‑form patients, the homozygous c.985_987delTACinsAA (p.Y329Kfs*90) mutation was most frequent (5/7). The remaining two (P1, P9) were compound heterozygotes for a frameshift (p.Y329Kfs*90 or c.775_776delAT, p.I259Hfs*16) and a missense mutation (c.286C>T, p.R96W, c.287G>A p.R96Q). The two partial‑form patients were also compound heterozygotes, carrying a missense mutation (c.1298T>C, p.L433S or c.995T>C, p.I332T) together with either a nonsense mutation (c.1218G>A, p.W406X) or a delins mutation (c.503_505delCCT, p.168_169delSFinsF).

The novel nonsense variant p.W406X truncates 103 C-terminal residues per homology modeling (**Figure 2**). It was tentatively classified as pathogenic under ACMG 2015 criteria with supporting evidence: PVS1 (null variant causing loss of function), PM2 (extremely low population frequency), PM3 (trans heterozygous state in a partial 17-OHD patient), PM4 (structural modeling) (9). Nevertheless, the 2018 ACMG supplementary guidelines advise restrained use of PVS1 for C-terminal truncants (10). While the substrate-binding I helix remains intact in the truncated product, the conserved heme-coordinating cysteine beyond residue 405 is eliminated. Without *in vitro* functional assays to measure residual enzymatic activity, complete loss of catalytic function cannot be validated solely by homology modeling.

**Figure 2:**
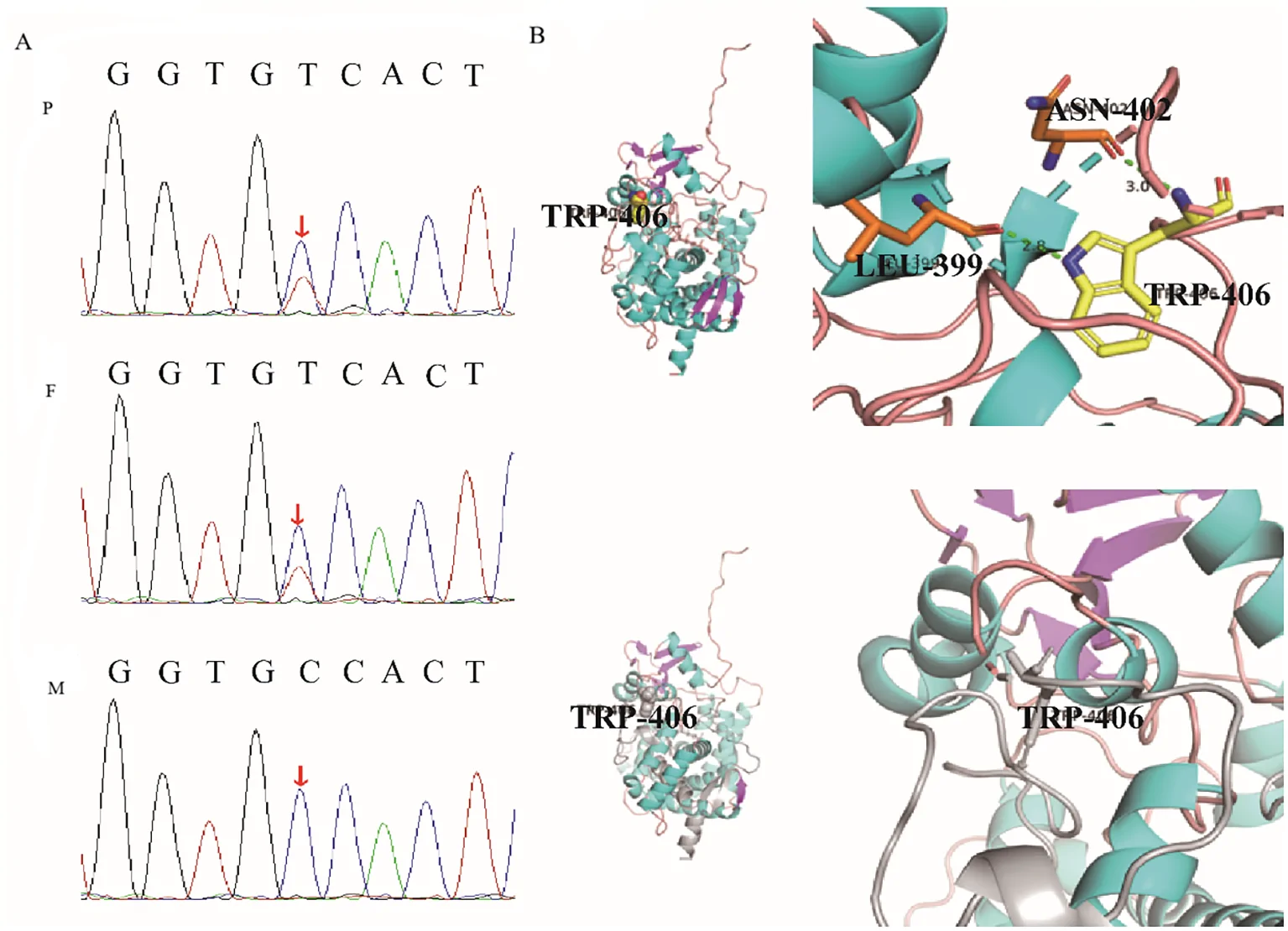
Parental identification and structural analysis of the novel p.W406X mutation. **(A)** The proband P7 and her father carried the novel p.W406X mutation. The mutated nucleotide is indicated by the red arrow. **(B)** Homology modeling of wild-type **(A, B)** and mutant **(A, B)** CYP17A1 protein. The p.W406X mutation introduces a premature stop codon, resulting in truncation of 103 C-terminal amino acids and predicted disruption of protein structure. P, proband; F, father, M: mother.

Whole‑exome sequencing of patient P6 and her parents revealed that both P6 and her mother carried a known *MYBPC3* mutation, c.1000G>A, p.E334K. Additionally, both P6 and her father carried variants in *ACTN2* (c.892C>T, p.R298C) and *TTN* (c.15488G>A, p.R5163H) (**Figure 3A**). All three variants were classified as variants of uncertain significance (VUS) according to ACMG criteria.

**Figure 3:**
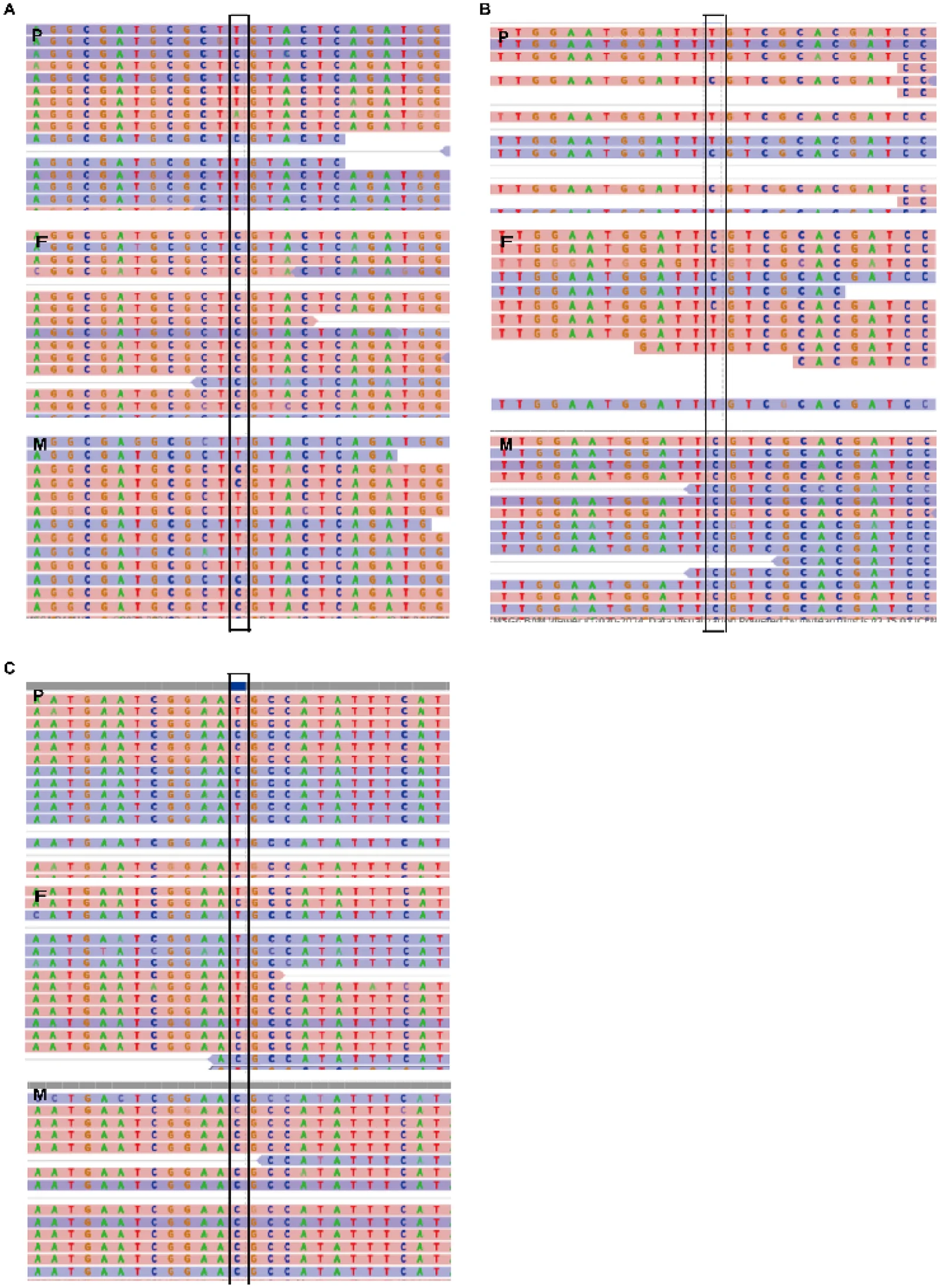
Genetic variants associated with LVNC in the pedigree of Patient P6. **(A)** *MYBPC3* variant c.1000G>A (p.E334K) present in the proband and her mother. **(B)** *ACTN2* variant c.892C>T (p.R298C) present in the proband and her father. **(C)** *TTN* variant c.15488G>A (p.R5163H) present in the proband and her father. The mutated nucleotide is indicated by the black box. P: proband, F: father, M: mother.

The study was approved by the institutional ethics committee, and written informed consent was obtained from all patients and/or guardians.

## Discussion

This systematic analysis of nine patients with 17-OHD reports, for the first time, an association of 17-OHD with TART and sensorineural hearing loss, and identifies a novel pathogenic mutation, p.W406X.

17-hydroxylase deficiency leads to hypocortisolism and elevated ACTH, resulting in increased 11‑DOC and corticosterone. These intermediates possess weak glucocorticoid activity (11), which may explain the absence of adrenal crisis despite low cortisol in most patients. Cases P7 and P9 both had elevated 11‑DOC and corticosterone without hypertension, aligning with reports that 10%–15% of 17-OHD patients are normotensive (12–14). A recent meta‑analysis showed no correlation between 11‑DOC and hypokalemia and noted a lower hypertension incidence in carriers of non‑null variants, with risk increasing with overall genotypic severity (11), suggesting additional regulatory factors.

While 46,XX 17-OHD patients may develop ovarian cysts (14–16), our two complete-type cases exhibited bilateral ovarian atrophy, low AMH, and elevated FSH, indicative of primary ovarian dysgenesis. Estrogen is vital for follicle maintenance, and complete CYP17A1 dysfunction eliminates fetal ovarian estrogen synthesis required to build primordial follicle reserves (17). However, aromatase-knockout mouse studies reveal fetal estrogen insufficiency alone cannot exhaust intrauterine follicles; severe atresia arises post-pubertally (18). We hypothesize the patients’ ovarian atrophy arises from synergistic prenatal hypoestrogenic injury and sustained pubertal endocrine dysfunction. *In utero* estrogen deficiency reduces primordial follicle stockpiles, whereas postnatal hypergonadotropism and chronic estrogen deprivation drive progressive follicular atresia and stromal fibrosis, ultimately lowering AMH and triggering ovarian atrophy (19).

Our nine-patient cohort revealed a tentative genotype–phenotype pattern: biallelic truncating loss-of-function variants (p.Y329Kfs^*^90, p.I259Hfs*16, p.R96W/Q) correlated with complete 17-OHD. The homozygous p.Y329Kfs^*^90 variant predominates in Chinese patients, consistent with a founder effect (2). Homozygous p.L433S was previously identified in a patient with hypospadias and penile dysplasia (20); our carrier P5 also presented with atypical genitalia, implying residual enzymatic activity sustaining minimal fetal androgen synthesis. The novel nonsense variant p.W406X truncates 103 C-terminal residues, deleting critical domains including the heme-binding region. Though c.1435_1438dupATCC (p.P480Hfs^*^27) induces a shorter frameshift, it fully abrogates enzyme activity across multiple pedigrees (21–23), supporting loss of function for p.W406X. Its trans counterpart c.995T>C (p.I332T) retains ~25% 17α-hydroxylase and ~10% 17,20-lyase activity and occurs in partial 17-OHD (1), confirming partial forms typically carry hypomorphic missense variants.

Data on gonadal malignancy risk in 17-OHD remain scarce, and guidelines for prophylactic gonadectomy lack consensus. Rare germ cell tumors in 46,XX patients and Leydig/Sertoli cell neoplasms in 46,XY individuals have been documented (24–26). All seven 46,XY patients in this cohort were raised as female and received gonadectomy; no malignant lesions were detected histologically. Patient P6 harbored focal steroidogenic cell nests within the dysplastic left testis; the nests were morphologically consistent with TART, which is prevalent in 21-hydroxylase deficiency (up to 94%) but rarely reported in 17-OHD. These lesions originate from multipotent embryonic progenitors, produce steroids, and co-express adrenal and testicular transcripts. Uncontrolled chronic elevation of ACTH and LH before hydrocortisone replacement likely drove lesion proliferation in P6 (27, 28). Hematoxylin-eosin staining cannot reliably distinguish TART from gonadotropin-induced Leydig cell hyperplasia; adrenal-specific immunohistochemistry (CYP11B2, calretinin) is required for definitive discrimination.

Left ventricular non-compaction (LVNC) features excessive ventricular trabeculation and deep intertrabecular recesses, stemming from failed embryonic myocardial compaction and linked to pathogenic sarcomeric variants in MYBPC3, TTN, and ACTN2 (29, 30). Patient P6 presented with rare dual diagnoses of 17-OHD and LVNC. The maternally inherited pathogenic MYBPC3 variant detected here is well-established in hypertrophic cardiomyopathy cohorts (31–33), whereas paternal TTN and ACTN2 variants are classified as variants of uncertain significance (VUS). No published evidence supports direct molecular or pathological crosstalk between *CYP17A1*-mediated steroidogenic defects and embryonic myocardial development. We therefore infer that the two diseases originate from distinct pathogenic pathways.

Two patients developed unilateral sensorineural hearing loss. This manifestation may be incidental or linked to endogenous CYP17A1 expression in cochlear tissue. Local steroid synthesis in cochlear hair cells and the stria vascularis sustains auditory homeostasis (34, 35). Complete loss of CYP17A1 enzymatic activity impairs intracochlear sex steroid and cortisol production, potentially inducing hair cell apoptosis and structural damage to the stria vascularis (36). In addition, chronic hypokalemia and hypertension secondary to 17-OHD impair inner ear microcirculation and aggravate hearing dysfunction (37). Larger cohorts are required to verify this mechanistic hypothesis.

This study has two main limitations. First, it is a single-center retrospective analysis of only nine patients with 17-OHD, without an integrated meta-analysis or a systematic summary of global case data. The small sample size prevented formal statistical validation of genotype–phenotype correlations; all observed links between CYP17A1 variants and clinical phenotypes are merely descriptive and hypothesis-generating, and cannot be extrapolated to the broader 17-OHD population. Large-scale multicenter cohorts are needed to confirm these preliminary associations. Second, all gonadal histopathological data were extracted from archived paper records, and original tissue slides were unavailable for re-examination and standardized high-power microscopic evaluation across all cases.

## Conclusion

This study systematically analyzed the clinical, endocrine, and genetic profiles of nine Chinese patients with 17-OHD, further expanding the pathogenic mutational spectrum of CYP17A1. A novel nonsense variant p.W406X was identified in a patient with partial 17-OHD and tentatively classified as pathogenic according to the ACMG criteria. A descriptive trend linking genotype to clinical subtype was observed: within the cohort biallelic loss-of-function variants were associated with complete 17-OHD, whereas missense variants retaining residual enzymatic activity predominantly resulted in partial phenotypes. Notably, our cohort presented multiple atypical manifestations, including ovarian atrophy, unilateral sensorineural hearing loss, suspected TART-like steroidogenic cell nests in gonadal tissue, and coexisting left ventricular non-compaction cardiomyopathy in one individual. Given the limitations of its single-center retrospective design and small sample size, this study provides only preliminary findings. Further large-scale cohort studies and *in vitro* functional analyses are warranted to validate these results.

## Data Availability

The original contributions presented in the study are included in the article/supplementary material. Further inquiries can be directed to the corresponding authors.
